# Histone Chaperone Deficiency in Arabidopsis Plants Triggers Adaptive Epigenetic Changes in Histone Variants and Modifications

**DOI:** 10.1016/j.mcpro.2024.100795

**Published:** 2024-06-05

**Authors:** Michal Franek, Martina Nešpor Dadejová, Pavlína Pírek, Karolína Kryštofová, Tereza Dobisová, Zbyněk Zdráhal, Martina Dvořáčková, Gabriela Lochmanová

**Affiliations:** 1 Mendel Center for Plant Genomics and Proteomics, Central European Institute of Technology, Brno, Czech Republic; 2 National Centre for Biomolecular Research, Faculty of Science, Masaryk University, Brno, Czech Republic; 3 Labdeers, Boskovice, Czech Republic

**Keywords:** chromatin remodeling, histone chaperone complex, histone variants, post-translational modifications, Arabidopsis, mass spectrometry, immunochemistry

## Abstract

At the molecular scale, adaptive advantages during plant growth and development rely on modulation of gene expression, primarily provided by epigenetic machinery. One crucial part of this machinery is histone posttranslational modifications, which form a flexible system, driving transient changes in chromatin, and defining particular epigenetic states. Posttranslational modifications work in concert with replication-independent histone variants further adapted for transcriptional regulation and chromatin repair. However, little is known about how such complex regulatory pathways are orchestrated and interconnected in cells. In this work, we demonstrate the utility of mass spectrometry–based approaches to explore how different epigenetic layers interact in Arabidopsis mutants lacking certain histone chaperones. We show that defects in histone chaperone function (*e.g.*, chromatin assembly factor-1 or nucleosome assembly protein 1 mutations) translate into an altered epigenetic landscape, which aids the plant in mitigating internal instability. We observe changes in both the levels and distribution of H2A.W.7, altogether with partial repurposing of H3.3 and changes in the key repressive (H3K27me1/2) or euchromatic marks (H3K36me1/2). These shifts in the epigenetic profile serve as a compensatory mechanism in response to impaired integration of the H3.1 histone in the *fas1* mutants. Altogether, our findings suggest that maintaining genome stability involves a two-tiered approach. The first relies on flexible adjustments in histone marks, while the second level requires the assistance of chaperones for histone variant replacement.

## Highlights

• Mass spectrometry–based study of epigenetic changes in chaperone-deficient plants.
• Chaperone deficiency in Arabidopsis is primarily compensated by histone PTMs.
• Changes in histone variant ratios and distribution follows as a slower adaptation.
• Plant responses to genotoxic agents involve PTM changes, not histone replacement.

## Graphical Abstract

**Figure:**
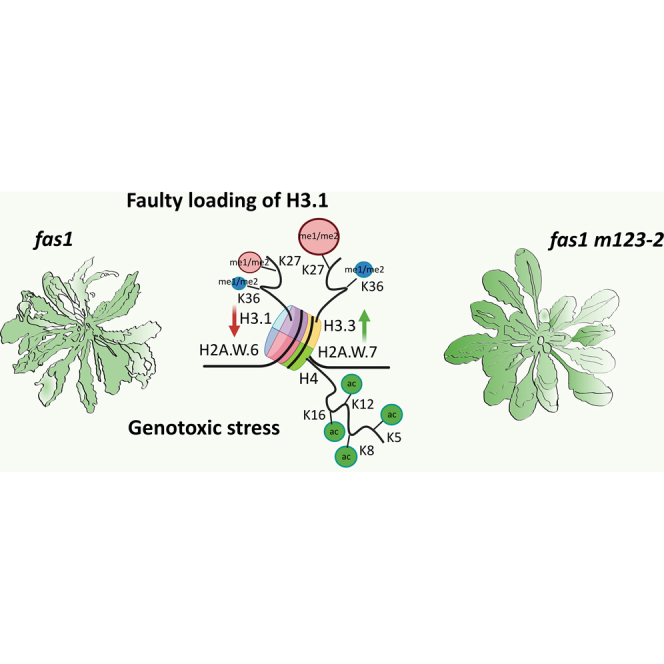

## In Brief

Using quantitative proteomics, we analyzed multiple layers of epigenetic changes in WT and histone chaperone-deficient Arabidopsis plants, even after genotoxic stress. We discriminated many histone variants and reconstructed the combinatorial pattern of histone PTMs. Rapid changes in the PTM profiles and slower changes in the histone variant ratios and histone variant distribution give a new insight in the plants’ ability to compensate the deficiencies in chromatin structure.

Chromatin assembly involving the incorporation of histones into DNA followed by posttranslational modifications (PTMs) is a fundamental regulatory mechanism that constitutes important parts of the epigenetic landscape, allowing both a flexible response to signaling cascades as well as heritable changes to the chromatin template that drive cell differentiation and function. They are involved in all key cellular physiological processes, including transcription, replication, repair, and cell division. Histones H1, H2, and H3 evolved different variants from their ancestral structure, with substantial functional differences and precisely regulated genome distribution.

H3 and H2A belong to the most diverse histone groups in Arabidopsis (1). The three types of H2A—H2A.X, H2A.W, and H2A.Z—have unique functions in the chromatin organization. H2A.Z is enriched in active euchromatic sites, absent from repeats and transposons (2), and essential for controlling developmentally regulated and stress response genes (3, 4). It modulates gene transcription near the transcriptional start site (5, 6), and its high levels in gene bodies are anticorrelated with gene activity (7, 8, 9). H2A.W regulates heterochromatin assembly, for example, chromocenter formation during early development and chromatin accessibility (10, 11, 12, 13). Recent studies indicate the participation of the H2A.W.7 subtype in DNA damage signaling in heterochromatin (14, 15), which complements the function of the predominant DNA damage signaling mediator H2A.X (13, 16). Histone H3 variants are known to functionally define centromeres (CenH3; (17, 18)), mark active genes (H3.3; (8, 19)), or associate with repetitive elements enriched in chromocenters (H3.1; (10)).

The mutual position of individual histone variants in chromatin is decisive for forming chromatin states with distinct properties. For example, H3.3 prevents the deposition of H1.1 on gene bodies, relaxes chromatin, and prevents H2A.Z from binding; therefore, *H3.3 kd* plants show increased H2A.Z levels (8). Another cooperation exists between H1, H3.1, and H2A.W that is required for the correct formation of chromocenters (10). Regardless of these local enrichments, most histone variants are present genome-wide. Thus, monitoring the various changes associated with their disbalance is generally challenging.

The assembly of canonical histones and histone variants into nucleosomes is mediated by multiple histone chaperones and nucleosome remodeling complexes. In our recent work (20, 21), we have focused on chromatin assembly factor-1 (CAF-1, consisting of fasciata 1 (FAS1), fasciata2 (FAS2), and multicopy suppressor of IRA1), which incorporates H3.1 during replication (22, 23). The set of cellular changes linked to dysfunctional H3 histone chaperone activity is severe and akin to the phenotype of *H3.1-* or *H3.3*-deficient plants, which (unlike chaperone mutants) show limited survival (8, 24). In the case of the *fas1*, the primary cause of varied defects is attributed to a major shift in the histone H3.3 to H3.1 ratio (20) translating into a more open chromatin configuration, for example, mislocalization of H3.3 (25), lower occupancy of the repressive mark H3K9me2 at repetitive elements (10), or high levels of the activating H3K4me3 mark at the defense genes (26). The local imbalance of histone variants and the redistribution of associated histone modifications cause defective formation of condensed chromocenters, loss of telomeres and *45S rRNA* genes (*rDNA*) (10, 27, 28), the increased hypersensitivity to genotoxic stress, and occurrence of damage sites (21, 29). Consequences of these defects are detectable as gene duplications and/or gene activation leading to transcriptional reprogramming (20, 29, 30).

Plants probably have the largest ability to compensate for the deficiencies of varied chromatin remodeling complexes (31, 32, 33). In *fas1*, severe phenotypic defects are ameliorated by disruption of nucleosome assembly protein 1 (NAP1; 1–3), a protein complex involved in the loading of H2A/H2B dimers (20, 34). The improved plant fitness, chromatin stability, resilience to DNA damaging agents, and significantly restored H3.1: H3.3 balance in *fas1 nap1;1 nap1;2 nap1;3* line (hereafter called *fas1 m123-2*) represent one example of histone chaperone network plasticity.

Changes in H3.1: H3.3 abundance open a question of how this imbalance affects the global level of related PTMs. While some modification sites are conserved between canonical H3 histones and variants (*e.g.*, H3K4me3 demarcating promoters of transcribed genes fulfill the same role on H3.1 and H3.3), others vary (H3K27 and H3K36 residues (35, 36). H3K27me1 is deposited during replication and involves the presence of CAF-1, proliferating cell nuclear antigen, and Arabidopsis trithorax-related protein 5 and 6 (ATXR5/6) that form one complex (24). ATXR5/6 selectively maintains K27me1 at H3.1, which is therefore reduced in *fas2* plants (37). Methylated H3K36 predominates at transcriptionally active regions, correlates with H3.3 enrichment, and antagonizes the H3K27me3 (38, 39). Evidence suggests functional interplay between individual histone modifications, a concept known as the histone code (40, 41). This has also been shown in Arabidopsis, where the ATXR5/6 histone methyltransferase discerns the methylation status of the H3K4 position, which in turn influences the deposition of H3K27me1 (42).

Due to the high level of complexity in the epigenetic regulation of cellular processes and the relatively low sensitivity of commonly used methods, the multiple layers of epigenetic changes arising from histone chaperone dysfunction have not been extensively studied in a mutant background. A detailed mapping of the variety of histone modifications is difficult to perform with techniques based on antibody detection. Chromatin immunoprecipitation techniques are essential for studying epigenetic modifications in the genomic context but are limited in the analysis of the co-occurrence of distinct histone variant/modification combinations (43). In this study, we took advantage of mass spectrometry (MS), which is unique in offering a comprehensive view of histone modifications, with the possibility to distinguish modifications on histone variants in peptide fragments with different amino acid sequences. This is especially relevant when studying plant mutants that exhibit complex cellular changes (*e.g.*, *fas1/fas2*) or studying cellular physiological processes that involve radical changes to the chromatin template. In our previous work, MS coupled with specific sample preparation techniques for plant histones (44) helped us to uncover changes in the H3.1: H3.3 ratio associated with the deficiency of CAF-1 (20).

Here, we applied a proteomic approach to discriminate histone variants and reconstruct the complex combinatorial pattern of histone PTMs in WT and histone chaperone loss-of-function mutants. We found that incorrect chromatin assembly in *fas1* translates into changes in the K36 and K27 methylation patterns in H3, altered ratio of H2A.W variants, and accumulation of H2A.W.7 in the nucleolar chromocenters. Based on the comparison of *fas1* and *fas1 m123*-*2*, we propose that these changes in chromatin structure are the consequence of compensatory mechanisms activated by CAF-1 dysfunction to maintain the functionality of individual chromatin domains. Furthermore, we revealed that treatment with genotoxic agents leads to lower H3K36 methylation levels on H3.3 in WT and *fas1* plants. On the contrary, concomitant minor shifts in H4 pan-acetylation observed in WT were not detected in *fas1*, which shows easier chromatin accessibility for DNA repair machinery (21, 27).

## Experimental Procedures

### Experimental Design and Statistical Rationale

This study was designed with the following three objectives. First, to monitor alterations in the levels and distribution of H2A histone variants in histone chaperone-deficient Arabidopsis mutant plants. Second, to evaluate changes in histone H3 methylation landscape resulting from CAF-1 histone chaperone dysfunction. Third, to evaluate changes in histone PTM status in WT and histone chaperone-deficient Arabidopsis plants upon exposure to the genotoxic agent zeocin.

Twelve-day-old seedlings of WT and mutant Arabidopsis plants (*fas1*, *fas1 m123-2*, *m123-2*) were used for histone extraction. The number of biological replicates was set to six for each condition. Histone extracts were derivatized with propionic anhydride. In total, 48 samples of WT and histone chaperone-deficient plants (with and without zeocin treatment) were subjected to LC-MS/MS analysis in data-dependent acquisition mode. Raw data were searched in Mascot search engine through Proteome Discoverer 2.2 software (https://www.matrixscience.com/) with the settings described below; threshold ion score (Mascot search engine) for acceptable peptide identification was 30, fragment match threshold for annotation was set to intensity of 10^4^. Identifications of selected histone peptides were manually verified and quantified based on the peak areas derived from the extracted ion chromatograms (EICs) using Skyline 20.1 software (https://skyline.ms/project/home/software/Skyline/begin.view), including identification alignment across the raw files based on retention time and *m/z*. The KNIME analytics platform was used for quantitative statistical analysis. No outliers were detected in the datasets. The detailed statistical rationale is described below.

### Plant Material and Zeocin Treatment

*Arabidopsis thaliana* seeds were sterilized (75% and 96% ethanol, 5 min each) and plated on half-strength agar Murashige and Skoog medium with 1% sucrose. A suction tweezer (GentleGrab, Labdeers) was used for the manipulation with the seeds. After 1-day stratification (4 °C/dark), plates were transferred to the growth chamber and grown for up to 1 week under long day conditions (16 h light – 21 °C/8 h dark – 19 °C/50–60% relative humidity). Seven-day-old seedlings were used for nuclei isolation for immunocytochemistry and protoplast isolation, while 12-day-old seedlings were used for bulk nuclei isolation on sucrose gradients for MS, as specified below. For the induction of genotoxic stress for MS analysis, plates with seedlings were treated with 20 μg/ml zeocin for 2 h. For long term induction of genotoxic stress, the seedlings were stratified for 3 days, grown for 3 days on MS plates and then transferred to MS plates with 20 μg/ml zeocin for 7 days.

### Nuclei Isolation on Sucrose for MS

A total of 0.6 g of 12-day-old seedlings were ground in liquid nitrogen into a fine powder, then resuspended in 20 ml of ice-cold nucleus isolation buffer, 10 mM Tris-Cl, 0.1 mM PMSF, 400 mM sucrose, 0.033% β-mercaptoethanol, and 1× protease inhibitors (Serva M307). Samples were left rotating for 10 min at 4 °C. Samples were filtered through a nylon mesh into an ice-cold tube and spun for 10 min at 2880*g* at 4 °C. Pellet was resuspended in 1 ml of ice-cold isolation buffer B (10 mM Tris-Cl, 0.1 mM PMSF, 250 mM sucrose, 1% Triton-X, 10 mM MgCl_2_, 1× protease inhibitor cocktail, 40 mM sodium butyrate) and centrifuged for 10 min at 12,000*g* at 4 °C. Pellet was resuspended in 300 μl of isolation buffer C (10 mM Tris-Cl, 1.7 M sucrose, 2 mM MgCl_2_, 0.15% Triton-X) supplemented with PTM inhibitors (1× protease inhibitor cocktail, #P9599, Sigma, 0.1 mM PMSF, and 40 mM sodium butyrate) and then overlaid on 1.5 ml of isolation buffer C in a new tube. Samples were centrifuged for 45 min at 16,000*g* at 4 °C, and pellets were further processed as described below.

### Histone Extraction and Derivatization

Histone extraction and derivatization were performed as described previously (20, 44, 45). Briefly, washed nuclei were incubated in nuclei lysis buffer (50 mM Tris–HCl, 100 mM NaCl, 3 mM EDTA, 1% 3-[(3-cholamidopropyl)dimethylammonio]-1-propanesulfonate) supplemented with PTM inhibitors (0.1 mM PMSF, 40 mM sodium butyrate, and 10 μl/ml protease inhibitor cocktail (P9599, Merck Millipore) for 1 h on ice. Histones were extracted from released chromatin into 0.2 M H_2_SO_4_. Plant histone extract in sulfuric acid was neutralized with NH_4_OH and subjected to a double round of propionic anhydride derivatization. Ten microliters of propionylation reagent (1:3 mixture of propionic anhydride and acetonitrile (MeCN); both from Sigma-Aldrich) were added to the samples and incubated in a thermomixer (37 °C, 700 rpm, 20 min). The sample volumes were reduced in a vacuum concentrator (Thermo Fisher Scientific) to 5 μl. For the second round of propionylation, samples were diluted with 50% (v/v) MeCN to 20 μl and propionylation was carried out using the same protocol. Samples were diluted with 300 μl of 8 M urea (pH 8.5), placed in a YM-10 Microcon filter unit (Merck Millipore), centrifuged (45 min, 14,000*g*, 25 °C), washed two times with 200 μl of 8 M urea, and three times with 100 μl of 100 mM ammonium bicarbonate (ABC; 45 min, 14,000*g*, 25 °C), followed by clean-up with 8 M urea (pH 8.5) on a YM-10 Microcon filter unit (Merck Millipore). After three washes with 100 μl of 100 mM ABC (45 min, 14,000*g*, 25 °C), 400 ng of SOLu-trypsin dimethylated (Sigma-Aldrich) diluted in 50 μl of 100 mM ABC was added. Digestion was carried out overnight at 37 °C. The digest was collected by centrifugation (10 min, 14,000*g*, 25 °C), subjected to two additional washes with 50 μl of 100 mM ABC, and concentrated the vacuum concentrator to a volume of ∼20 μl. One microliter of NH_4_OH and 5 μl of the propionylation reagent prepared by mixing propionic anhydride with MeCN in a 1:3 ratio were added. The pH was adjusted to 8 to 9 with NH_4_OH, samples were incubated in thermomixer at 37 °C at 700 rpm for 20 min and then the volume was reduced in the vacuum concentrator to 5 μl. For the second round of propionylation, samples were diluted with 50% (v/v) MeCN to a volume of 20 μl and the process was carried out using the same protocol. The samples were diluted with 0.1% formic acid to a volume of 100 μl. Labeled histones were desalted using a Hypersep SpinTip C-18 column (Thermo Fisher Scientific).

### LC-MS/MS, Database Search, and MS-Data Evaluation

Propionylated peptides were measured using LC-MS/MS. The samples were spiked with the iRT-C18 reference peptides (iRT-Kit, #Ki-3002-1, Biognosys). The LC-MS/MS equipment consisted of an RSLCnano system, equipped with an C18 PepMap100 trap column (5 μm particles, 300 μm × 5 mm; Thermo Fisher Scientific), and an Acclaim PepMap100 C18 analytical column (3 μm particles, 75 μm × 500 mm; Thermo Fisher Scientific), coupled to an Orbitrap Lumos Tribrid spectrometer (Thermo Fisher Scientific) equipped with a Digital PicoView 550 ion source (Scientific Instrument Services), and active background ion reduction device. Prior to LC separation, tryptic digests were online concentrated on a trap column. The mobile phase consisted of 0.1% formic acid in water (A) and 0.1% formic acid in 80% acetonitrile (B). Peptide elution was done by 85 min long nonlinear gradient separation at 300 nl.min-1 with increasing content of mobile phase B: 5 to 25% B for the first 20 min, 25 to 29% B for 20 to 30 min, 29 to 32% B for 30 to 40 min, 32 to 38% for 40 to 55 min, 38 to 50% B for 55 to 75 min, and 50 to 85% B for the last 10 min. The analytical column outlet was directly connected to the ion source of the MS. MS data were acquired in data-dependent mode using survey scan in *m/z* range 350 to 2000 at a resolution of 60,000 (200 *m/z*) with target value of 4 × 10^5^ and maximum injection time 54 ms. Precursors with charge state from 2+ to 7+ and intensity above 1 × 10^4^ underwent higher-energy collisional dissociation fragmentation with normalized collision energy of 30. Once fragmented, precursors were excluded for 60 s prior next fragmentation. Precursor isolation was performed in quadrupole with isolation window 1.6 *m/z*. Tandem mass spectra were obtained for ions with *m/z* value at least 110 using resolution 30,000 (200 *m/z*). Ions were accumulated for a target value of 5 × 10^4^ or 500 ms injection time. Cycle time between master scans was 3 s.

The RAW mass spectrometric data files were analyzed using Proteome Discoverer software (Thermo Fisher Scientific, version 2.2) with an in-house Mascot search engine (Matrixscience, version 2.6.2) to compare acquired spectra with entries in the UniProtKB *Arabidopsis thaliana* protein database (version 2020_04; 27463 protein sequences; downloaded from ftp://ftp.uniprot.org/pub/databases/uniprot/current_release/knowledgebase/reference_proteomes/Eukaryota/UP000006548_3702.fasta.gz), cRAP contaminant database (downloaded from http://www.thegpm.org/crap/) and in-house AT-histone database (version 2020_01; 61 protein sequences). Mass tolerances for peptides and tandem mass spectrometry (MS/MS) fragments were 7 ppm and 0.03 Da, respectively. Semi-Arg-C for enzyme specificity allowing up to two missed cleavages was set. For searches against cRAP, the variable modification settings were oxidation (M), deamidation (N, Q), acetylation (K, protein), and propionylation (K, N-term, S, T, Y), for UniProtKB *Arabidopsis thaliana* databases they were acetylation (K, protein) and propionylation (K, N-term), while for histone database searches they were acetylation (K, protein N-term), methylation (K, R), dimethylation (K), trimethylation (K), phosphorylation (S, T), and propionylation (K, N-term, S, T, Y). No fixed modification was set. Peptides identified using the UniProtKB *Arabidopsis thaliana* protein database were refined by Percolator (Cn <0.05, false discovery rate <0.01). Peptides identified using the in-house AT-histone database were refined by fixed-value peptide-spectrum match validator (delta Cn < 0.05), followed by filtering highly confident peptides using Mascot parameters set to Rank 1, expectation value <0.01, and ion score ≥30 were considered.

The peak area corresponding to each precursor ion was calculated from the EICs using the precursor ions area detector node. Selected histone peptide identifications were manually verified and quantified from the peak areas derived from the EICs using Skyline 20.1 software, including identification alignment across the raw files based on retention time and *m/z*. The KNIME analytics platform was used for quantitative statistical analysis (46).

The relative abundance of a particular modified peptide form was calculated from the ratio of each precursor peak area to the total area of the respective peptide sequence. The relative abundance of a particular histone sequence subvariant (*e.g.*, H2A.Z, H2A.X, or H2A.W) was calculated as the ratio of the averaged area of the precursor peaks of its unique peptides to the total area of these variants. The peak areas corresponding to posttranslationally modified forms of individual histone peptide were treated as compositions and Aitchison’s methodology based on log-ratios was applied in the statistical evaluation. First, the missing values were imputed by iterative least trimmed squares regression (ilr), and areas were transformed to relative abundances (percentages). In order to evaluate global acetylation or methylation, acetylated and nonacetylated forms, and analogically methylated and nonmethylated forms of each peptide were amalgamated. These amalgamated abundances were then ilr-transformed and compared by Hotelling’s T2 test to globally assess the differences in their distribution. For comparison of all individual peptide forms and histone variants, for each peptide, the log2 ratio of relative abundance of one form to the sum of relative abundances of all other forms (alr-transformation of a two-part composition) was calculated. The *t* test was applied to assess the difference in each individual form between the two conditions (*i.e.*, WT *versus* WT-Z or *fas1 versus fas1*-Z). For multiple comparison (*i.e.*, WT *versus fas1 versus fas1 m123-2 versus m123-2*), one-way ANOVA with post hoc Tukey’s honest significant difference test correction was applied. Note that in compositional data, the relative abundances of individual parts were not directly comparable due to the constant sum constraint leading to a spurious negative correlation. The data analysis was performed in R version 3.6.3 (R core team (2020). R: A language and environment for statistical computing. R foundation for statistical computing. URL https://www.R-project.org/) using the compositions and Hotelling R packages for ilr and alr transformations and Hotelling T2 test, respectively, (K. Gerald van den Boogaart, Raimon Tolosana-Delgado and Matevz Bren (2020). Compositions: compositional data analysis. R package version 1.40 to 5. https://CRAN.R-project.org/package=compositions; James Curran (2018). Hotelling: Hotelling’s T∧2 Test and Variants. R package version 1.0 to 5. https://CRAN.R-project.org/package=Hotelling).

### Immunocytochemistry

Nuclei isolation for immunochemistry and immunolabeling were performed as outlined in (47). Briefly, 7-day-old seedlings were chopped with a razor blade in the nucleus isolation buffer (500 mM sucrose, 100 mM KCl, 10 mM Tris-Cl, 10 mM EDTA, 4 mM spermidine, 1 mM spermine). The suspension of nuclei was filtered through 50 μm and 30 μm CellTrics filters and centrifuged at 2000*g*, 10 min, 4 °C. The pellet containing nuclei was resuspended in nucleus isolation buffer, spotted on Superfrost glass slides and left to dry at 4 °C. For immunolabeling, the nuclei were permeabilized in 0.5% Triton-X in 1× PBS and then blocked in 5% bovine serum albumin in 1× phosphate buffered saline with 0.05% Tween 20 and incubated with a primary antibody overnight (anti H3K9me2 – #ab1220 Abcam; anti H3K27me1 – #07-448 Millipore; anti H3K4me3 – #ab8580 Abcam; anti-H4 pan-acetylation – #17-211 Millipore) at 4 °C. After 3× wash in 1× phosphate buffered saline with 0.05% Tween 20, the samples were incubated with the secondary antibodies (anti-rabbit Alexa Fluor 488 #A-11008, Invitrogen; and anti-mouse Alexa Fluor 594 #A-11005, Invitrogen), washed, and mounted in 4′,6-diamidino-2-phenylindole + Vectashield (Vector Laboratories).

### Cloning and Protoplast Transformation

Cloning and bimolecular fluorescence complementation (BiFC) was performed as shown previously (20, 48). Entry clone containing complete coding sequence (20) of NAP1;1 gene (AT4G26110) was synthesized from total RNA of 10-day-old seedlings using M-MuLV reverse transcriptase (#M0253, New England Biolabs) and Phusion HF DNA polymerase (#M0530, New England Biolabs) with specific primers (20). Plasmid vectors containing complete coding sequence of histone variants H2A.Z.8 (AT2G38810.1), H2A.W.7 (AT5G27670.1), and H2A.Z.11 (AT3G54560.1) were obtained from the Arabidopsis Biological Resource Center (The Ohio State University). All clones were recloned to Gateway compatible vectors pDONR207, pK7WGR2 (for localization experiments), pSAT1-nEYFP, and pSAT1-cEYFP (for BiFC) using BP and LR clonase (#11789013 and #11791019, Thermo Fisher Scientific). For detection of transformation efficiency, the protoplasts were cotransfected with the plasmid expressing mRFP and the nuclear localization signal of the VirD2 protein of *Arabidopsis tumefaciens* (mRFP-VirD2NLS) (49). Plasmid DNA was isolated with the Nuclebond Xtra Midi kit (#740410, MACHEREY-NAGEL). All procedures were carried out in accordance with manufacturer’s protocols. Protoplast isolation and transformation with plasmid vectors were performed as described in (50). Briefly, 7-day-old seedlings were partially homogenized in a Petri dish and incubated in the enzymatic solution (3 h on a shaker) consisting of 0.4% cellulase, 0.04% pectolyase, and 0.2% macerozyme (#C8001, #P8004, #M8002, Duchefa Biochemie) to digest the cell wall and release protoplasts. The protoplast solution was then filtered through a 70 μm sterile mesh, centrifuged at 200*g*, 4 min, 4 °C, and washed in W5 solution (154 mM NaCl, 125 mM CaCl2, 5 mM KCl, 5 mM glucose, and 2 mM 2-(N-morpholino)ethanesulfonic acid, pH = 5.7). Transformation of protoplasts with H2A.Z.8-mRFP, H2A.W.7-mRFP, NAP1;1-cYFP, H2A.Z.8-nYFP, and H2A.Z.11-nYFP was performed by the addition of 50% polyethylene glycol for 25 min, which was followed by 2× wash in W5 solution and transfer of protoplasts into a 24-well plate coated with 1% bovine serum albumin. The analysis of H2A.W.7 distribution was performed in two independent biological replicates for the *fas1* mutants and three independent biological replicates for the WT, using the two-sided binomial test from the SciPy python library.

### Microscopy

Imaging of protoplasts was performed on the Zeiss LSM-800 and Zeiss LSM-880 confocal systems using a 63× plan-apochromat water objective. To analyze histone modifications, the samples were imaged with a 63× oil plan-apochromat objective on a confocal (LSM-800) and super-resolution system (Zeiss Elyra 7) using the lattice-structured illumination microscopy mode.

## Results

Recently, close interactions between NAP1 and FAS1 pathways have been demonstrated by both genomic and proteomic approaches, showing that many features of CAF-1 deficiency can be disrupted by knocking out *Nap1* genes (20). To further elucidate the nature of chromatin instability in Arabidopsis chaperone loss-of-function mutants, we performed a thorough comparison of the WT histone epigenetic pattern with that of *fas1*, *m123-2* (*i.e.*, *nap1;3-2* triple mutant), and *fas1 m123-2* (*i.e.*, *fas1-4 nap1;1 nap1;2 nap1;3-2* quadruple mutant; Supplemental Fig. S1*A*). In parallel, we monitored changes in histone mark levels following genotoxic-induced DNA damage in WT and *fas1* plants (Supplemental Fig. S1*B*). The main focus of the study was on H2A variants and the PTMs of the histones H3 and H4.

### Profiling of Histone Variants in Arabidopsis Histone Chaperone Mutants

Prompted by the fact that *fas1* plants display altered H3.1 and H3.3 ratio, which is partially restored in the *fas1 m123-2* mutant (20), and that the H2A.W expression is affected in *fas2* plants (10), we wondered how the altered ratio of H3 variants affects the H2A histone pools and PTMs present at H2, H3, and H4 histones. Using the previously generated MS datasets (20), we examined the relative levels of histone variants in the WT, *fas1*, *m123*-*2*, and *fas1 m123-2* plants. Based on the unique peptides in the amino acid sequence, we could distinguish 30 histone protein groups in WT and mutant plants, including those with a high percentage of similarity in their sequences. Specifically, we focused on H2A.W.6, H2A.W.7, H2A.Z.8, H2A.Z.9, H2A.Z.11, H2A.X.3 and H2A.X.5 variants, and modifications of H3.1, H3.3 and H4. We achieved high sequence coverage of all target proteins, which is a prerequisite for characterizing their overall modification status. The most complex PTM pattern involving acetylation and multistage methylation (monomethylation, dimethylation, or trimethylation) was found in histone H3, while variants of histones H2A (namely H2A.Z and H2A.1) and H4 were exclusively acetylated (Supplemental Fig. S2). We did not detect any PTMs in the H2A.W and H2A.X variants at the given sequence coverage and filter criteria used. Since the histones are extracted from isolated chromatin, the pool of “free” histone variants will be minimal and the observed changes predominantly reflect the chromatin-bound fraction. The complete list of the identified histone peptide forms with the description of the associated proteins, including both UniProt and phylogeny-based names recommended by (51) is enclosed in Supplemental Table S1.

### Alterations in the Levels of H2A.Z and H2A.X Variants Resulting from Chaperone Dysfunction

**Fig. 1:**
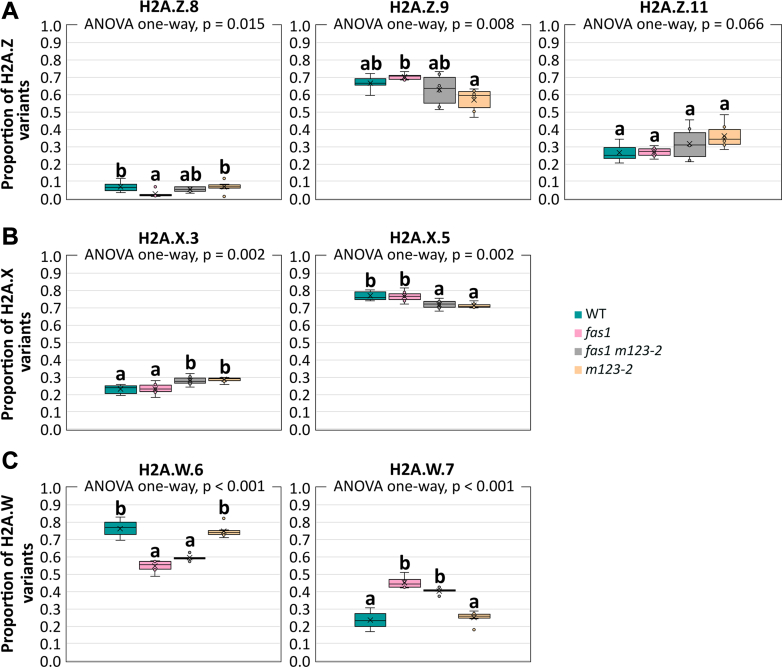
**The proportion of H2A variants is significantly affected inhistone chaperoneloss-of-functionmutant plants.***Box plots* of the H2A.Z (*A*), H2A.X (*B*), and H2A.W (*C*) proportion in WT and mutant plants obtained from MS data showing extremes, interquartile ranges, means, and medians (n = 6). For each histone variant, letters a and b indicate significant differences between WT, *fas1*, *fas1 m123*-*2*, and *m123-2* according to one-way ANOVA with post hoc Tukey’s HSD test, *p* < 0.05. FAS, fasciata; MS, mass spectrometry.

The H2A variants shape the epigenetic landscape and represent an important level of transcriptional regulation. As the H2A.Z levels rise upon H3.3 depletion (8), we wondered whether the increased relative H3.3 levels in *fas1* affect the abundance of H2A.Z or the balance of its variants. Our results revealed the difference in the proportion of H2A.Z histone variants in the WT; H2A.Z.9 (∼67% of all H2A.Z) was the predominant variant, while the H2A.Z.8 (∼7%) and H2A.Z.11 (∼25%) were much less abundant (Fig. 1*A*). We did not see the change in the overall level of H2A.Z variant in *fas1* compared to the WT (Supplemental Fig. S3). Subtle changes in the proportion of H2A.Z variants in *fas1* in terms of reduced level of low-abundant H2A.Z.8 (Fig. 1*A*) indicate that the relative increase in H3.3 levels does not substantially affect the H2A.Z pool. Interestingly, the level of H2A.Z.9 in the *m123-2* (depleted for *nap1–3* genes) was lower while the level of H2A.Z.11 showed an increased trend compared to WT (Fig. 1*A* and Supplemental Table S2). The role of NAP1 in the H2A.Z processing was previously shown for *Saccharomyces cerevisiae* (52, 53). In Arabidopsis, the function of NAP1-related proteins (NRP1 and NRP2) in relation to H2A.Z has been reported (54). Therefore, using the BiFC assay, we tested whether the plant NAP1 could interact with H2A.Z variants *in vivo*. Our results confirmed that NAP1 can bind H2A.Z in the cytoplasm, similarly to its interaction with H3 and H2A (20, 55), Supplemental Fig. S4, *A* and *B*. NAP1 binding to H2A.Z was not affected by *FAS1* deficiency, consistent with the normal distribution of H2A.Z variants observed in *fas1* line (Supplemental Fig. S4, *C* and *D*). We also assessed the H2A.Z.8 localization pattern in WT and *m123-2* root protoplasts, revealing a dispersed signal of H2A.Z.8 identical in WT and *m123-2* plants (Supplemental Fig. S4, *E* and *F*).

We next looked at the relative abundance of the H2A.X, which shows a genome-wide distribution in Arabidopsis and plays an important role in double-strand break signaling *in vivo* (13, 16). The H2A.X: H2A.W: H2A.Z ratio (Supplemental Fig. S3) and the ratio of H2A.X variants in *fas1* line—H2A.X.5 and H2A.X.3 (Fig. 1*B*)—remained comparable to WT, while moderately decreased H2A.X.5 was detectable in *fas1 m123-*2 and *m123-2* (Fig. 1*B*). Though H2A.X has been mostly investigated in connection to DNA repair, the changes observed in *fas1 m123-2* and *m123-2* may also relate to the alternative chromatin assembly that compensates for histone chaperone deficiency in these mutant plants.

### FAS1 Dysfunction Leads to the Increase in H2A.W.7 Variant Concomitant with its Redistribution in the Cell Nucleus

In the case of H2A.W, we were able to quantify two of the three variants H2A.W.6 and H2A.W.7; the H2A.W.12 was excluded from the quantification due to missing identification of unique peptides. Our data showed that the H2A.W.6 was dominant over H2A.W.7 in WT and *m123-2* (Fig. 1*C*). Curiously, the ratio of H2A.W.6: H2A.W.7 variants changed in the *fas1* and in *fas1 m123-2*, when compared to WT, with the *fas1 m123-2* showing an intermediate phenotype (Fig. 1*C* and Supplemental Table S2), similarly to the pattern of H3.1: H3.3 (20). Relative abundance of H2A.W (when compared to other studied histone variants) remained unchanged in CAF1-deficient mutants (Supplemental Fig. S3).

Considering the partially distinct functions of H2A.W.6 and H2A.W.7 in chromatin organization and DNA damage response (14) as well as the *fas1* line being prone to genotoxic lesions, we tested whether the changes in the levels of H2A.W.7 translate into changes in the histone distribution in cells. H2A.W.7 was previously shown to localize in the chromocenters in Arabidopsis roots *in vivo* and *in situ* by immunodetection (13, 14). Using the protoplast system and H2A.W.7-mRFP fusion, we assessed the localization pattern in protoplasts prepared from WT and *fas1* 7-day-old seedlings.

**Fig. 2:**
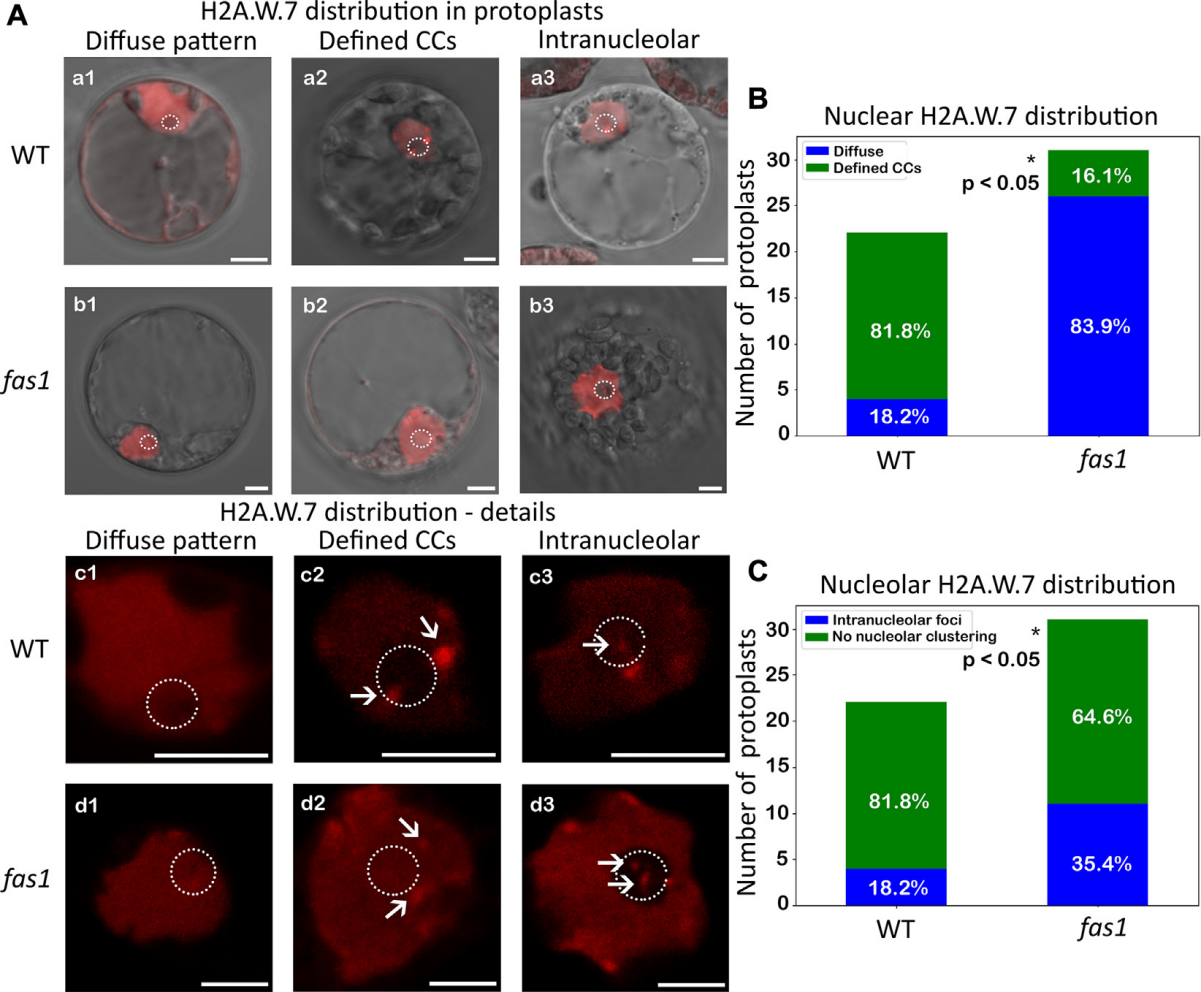
**Distribution of the H2A.W.7 histone variant in the nucleus of WT and *fas1* plants.***A*, distribution patterns of H2A.W.7-mRFP in the nuclei of WT (a, c) and *fas1* plants (b, d). Histone H2A.W.7 shows either a diffuse pattern (1) or a distribution with marked accumulation in chromocenters (2). Intranucleolar foci of H2A.W.7 occur rarely in WT and are more common in the *fas1* nuclei (3). *White arrows* indicate localization of H2A.W.7 in nuclear chromocenters in c2, d2 and intranucleolar foci in c3, d3. Statistical evaluation of the nuclear (*B*) and nucleolar (*C*) H2A.W.7 clustering, using two-sided binomial test with the Scipy python library, *p* < 0.05 (∗). Number of analysed nuclei was 22 for WT and 31 for *fas1*. Nucleoli are highlighted by a *white circle*. The scale bar represents 5 μm. FAS, fasciata.

In the WT, the majority (∼82%) of nuclei showed the expected localization pattern with H2A.W.7 accumulated to the well-defined foci (representing segments of repressive chromatin termed chromocenters; Fig. 2, *A* (a2, c2), and *B*) and about 18% nuclei showed a diffuse pattern without these prominent structures (Fig. 2, *A* (a1, c1) and *B*). Opposed to WT, the diffuse pattern was predominant in the *fas1* background (∼84%; Fig. 2, *A* (b1, d1) and *B*), with a small number of cells showing chromocenters (∼16%; Fig. 2, *A* (b2, d2) and *B*). Importantly, intranucleolar clusters of H2A.W.7 (Fig. 2*A* (a3–d3)) occurred more frequently in the *fas1* (∼35%, Fig. 2*C*) than in the WT (∼18%, Fig. 2*C*). These results may suggest increased DNA damage events occurring in the *rDNA* in *fas1*, consistent with previous results indicating higher occurrence of DNA breaks in ribosomal genes detected as γH2A.X foci (21). Alternatively, it may reflect inefficient H3.1 deposition changing the chromatin composition inside the nucleolus.

### Broad Changes in the Histone H3 Methylation Landscape Occur as a Result of the CAF-1 Histone Chaperone Dysfunction

**Fig. 3:**
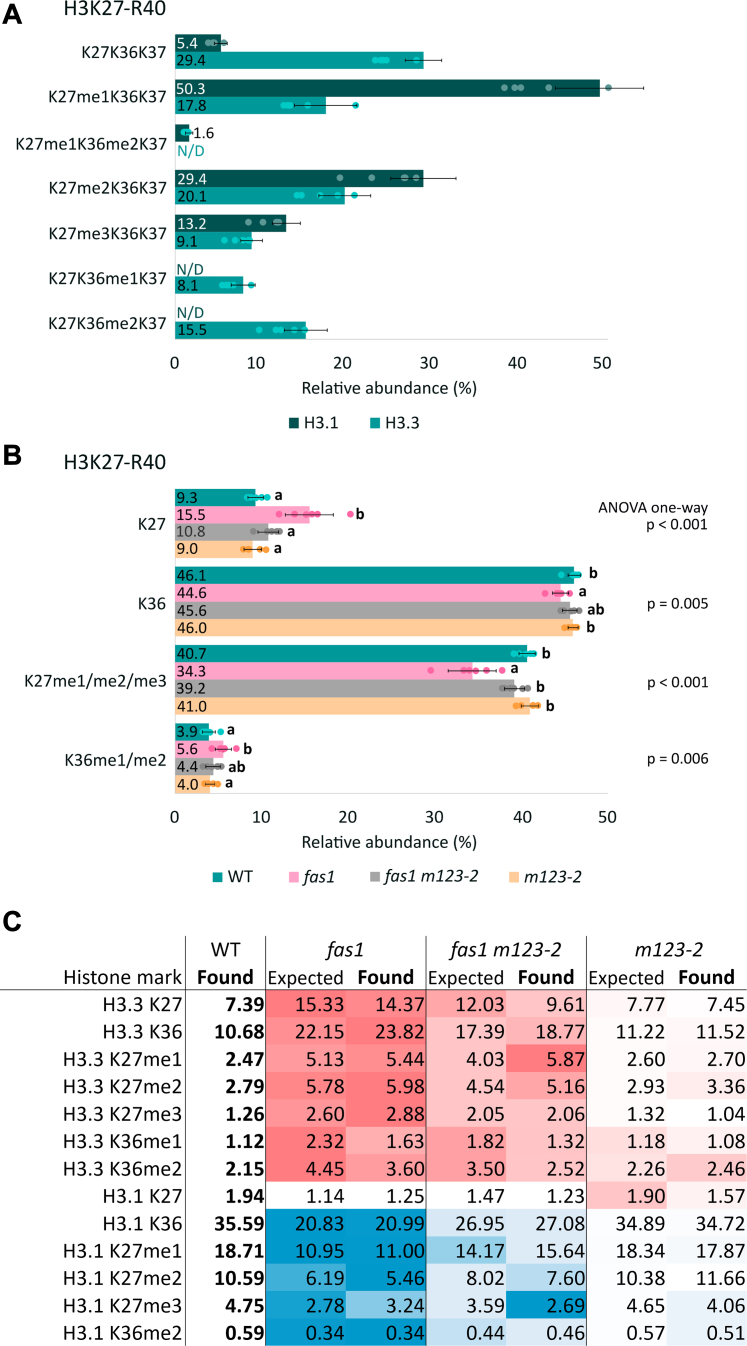
**Quantitative MS analysis disclosed distinct K27–R40 methylation profiles in H3.1 and H3.3 variants.***A*, relative abundance of H3.1K27–R40 and H3.3K27–R40 methylated forms in WT (sum of all peptide forms in each H3 variant equals 100%). *B*, relative abundance of K27 and K36 marks in H3K27–R40 sequences (histone marks of H3.1 and H3.3 were counted together; sum of histone marks in both histone variants equals 100%) in the WT and loss-of-function chaperone mutant plants. Numbers correspond to the mean values (n = 6) of precursor peak areas in percentages. For each histone form, letters a and b indicate significant differences between WT, *fas1*, *fas1 m123*-*2*, and *m123-2* according to one-way ANOVA with post hoc Tukey’s HSD test, *p* < 0.05. *C*, relative abundance of K27 and K36 marks in H3.1K27–R40 and H3.3K27–R40 sequences reflecting the H3.1: H3.3 ratio in WT and loss-of-function chaperone mutant plants (sum of histone marks in both H3 variants equals 100%). Column “Expected” shows the theoretical abundance calculated from WT values with respect to the H3.1: H3.3 ratio in a particular mutant, while experimental values obtained from MS data are presented in column “Found.” Colors in the heatmap reflect the increase (*red*) or decrease (*blue*) of histone mark levels in mutant plants compared to WT level (*white*). FAS, fasciata; HSD, honest significant difference; MS, mass spectrometry.

We have next focused on the H3 variants as these are the targets of the CAF-1 chaperone, and we hypothesized that the dysfunction of CAF-1 might trigger changes in the different modification profiles of H3.1 and H3.3. The amino acid sequence of H3.3 variant is nearly identical to H3.1 (97% identity); however, the variants can be distinguished in the mass spectrum due to the alanine/threonine replacement at the 31st position of the K27−R40 segment (**H3.1K27**SAP**A**TGGVKKPH**R40** and **H3.3K27**SAP**T**TGGVKKPH**R40**), resulting in a 30 Da mass shift. This peptide is methylated at two major positions, K27 and K36 with a variant-specific pattern (35, 36). While alanine 31 is decisive for selective K27me1 in H3.1 variant, K36me1/2/3 is predominant in H3.3 and not easily detected in H3.1 due to the low abundance. Detailed mapping of PTMs in WT plants revealed clear differences in the levels of methylation on the K27−R40 peptide of H3.1 and H3.3 (Fig. 3*A*). The majority of H3.1K27−R40 peptides were methylated (only ∼5% of nonmodified K27K36K37 was found, Fig. 3*A*) with predominant K27me1, whereas only ∼71% of methylated forms were present in H3.3K27−R40. In addition, methylated K36 was detected as a very low-abundant mark in H3.1 but more than 20% of K36 is occupied by methylation or dimethylation in H3.3 (Fig. 3*A*).

Since CAF-1 deficiency causes increased incorporation of the less methylated H3.3 variant in individual chaperone mutants, we wondered whether this will be reflected in the K27−R40 methylation pattern. The sum of methylated K27 and K36 marks in WT represents ∼41% and ∼4% of all histone H3 forms, respectively (Fig. 3*B*). We found that global methylation in *fas1* reached a lower level than WT as the sum of methylated K27 and K36 marks account for ∼34% and ∼6% of all histone H3 forms, respectively (Fig. 3*B* and Supplemental Table S3). Previously determined distinct H3.1: H3.3 ratio in *fas1* and WT (∼0.8: 1 and 3: 1, respectively; (20)) indicates that disrupted K27−R40 methylation profile in the *fas1* is mostly caused by reduced levels of H3.1 which account for high methylation level at K27.

In the case of K27 methylation, the *fas1* is similar to the *h3.1 kd* plants, where K27me1 and me3 are much less abundant; this is consistent with the previously observed pattern in *fas2* (24, 37). Considering the H3.1: H3.3 ratio in the mutant plants (20), we calculated the theoretical abundance of K27 and K36 methylated marks (as expected by a simple exchange of H3.1 for H3.3) and compared it to the experimental data. To our surprise, we detected a higher than expected level of H3.3K27 methylation together with a lower level of H3.3K36 methylation in the *fas1* and *fas1 m123-2* (Fig. 3*C*). Such fine-tuning of histone PTM profiles may imply adaptation of the mutants to the faulty loading of H3.1 onto chromatin and partial repurposing of the H3.3 histone variant (Fig. 3*C*). In the *fas1 m123-2*, the difference between the expected and experimentally obtained values was even more striking than in *fas1*. Next to H3.3K27me1, an increased level of K27me1 was observed also in H3.1. Altogether, our data show that the compensatory effect of the absent *Nap1;1 to 3* genes is manifested at the levels of both histone incorporation and PTMs, directing the global methylation status in *fas1 m123-2* toward that of the WT and the *m123-2* line (Fig. 3*B*).

No significant differences in the levels of posttranslationally modified peptides sharing the same amino acid sequence between the H3.1 and H3.3 variants (K9−R17 and K18−R26) and at the N-terminus of histone H4 (G4−R17) have been found between WT and chaperone mutants. Relative abundance and statistical evaluation of posttranslationally modified forms of histones H3.1, H3.3, and H4 are presented in Supplemental Table S3. Certain low-abundant marks identified by LC-MS/MS (*e.g.*, H3.3K36me3 and H3.3K36ac) were excluded from further quantification due to incomplete fragment ion series or low-quality EICs.

Since the detection of minor changes in the levels of histone modifications is generally not possible with immunolabelling due to its detection limit, we focused on the possible changes in the distribution of histone modifications in plant nuclei. As seen in Supplemental Fig. S5, we did not detect changes in the distribution of repressive (H3K9me2/H3K27me1; Supplemental Fig. S5, *A*–*D*) or activating histone modifications (H3K4me3/H4 pan-acetylation; Supplemental Fig. S5, *E*–*H*) between the WT and the *fas1* plants with malfunctioning key histone chaperones.

### Changes in Histone PTM Status upon Exposure to Genotoxic Stress

**Fig. 4:**
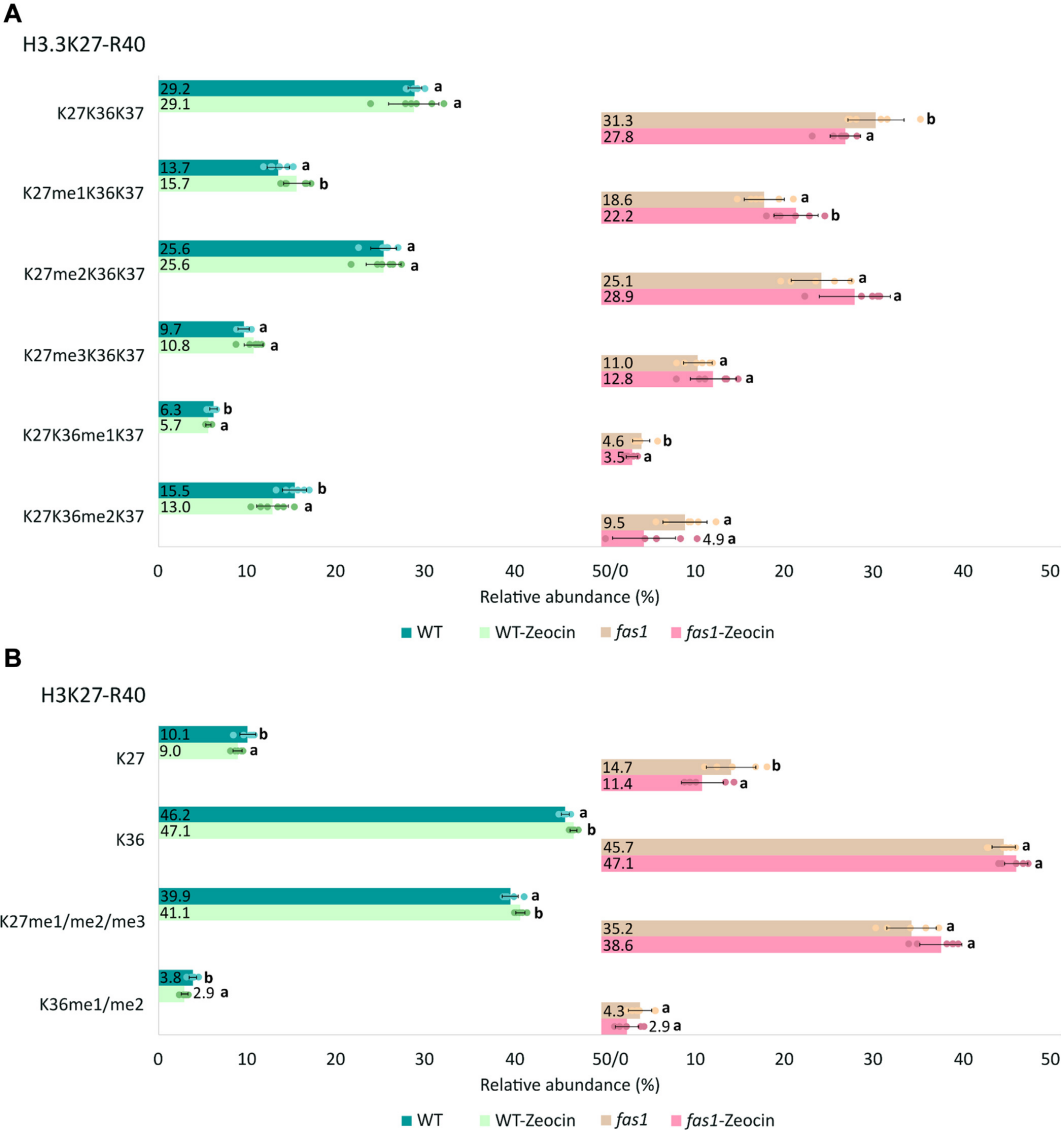
**The impact of zeocin treatment on K27–R40 methylation profiles in H3.1 and H3.3 variants.***A*, relative abundance of H3.3K27-R40 methylated forms after zeocin treatment in WT and loss-of-function CAF-1 mutant plants (sum of all peptide forms in H3.3 variant equals 100%). *B*, relative abundance of K27 and K36 marks in H3K27–R40 sequences (histone marks of H3.1 and H3.3 were counted together; sum of histone marks in both histone variants equals 100%) in WT and loss-of-function CAF-1 mutant plants after zeocin treatment. Numbers correspond to the mean values (n = 6) of precursor peak areas in percentages. For each histone form, letters a and b indicate significant differences between control (WT or *fas1*) and respective line treated with zeocin, according to the Student *t* test at *p* < 0.05. CAF-1, chromatin assembly factor-1; FAS, fasciata.

Since the repair of bulky DNA lesions or DNA breaks involves chromatin remodeling, which might be impaired in the chaperone mutants, we tested whether treatment with a clastogenic compound zeocin will alter the levels of selected histone marks based on previous data showing *fas1* hypersensitivity to zeocin (20). Histones in the *fas1* were found to be more susceptible to changes in the methylation status after zeocin treatment compared to WT. The levels of H3.3K27 monomethylated marks in *fas1* increased significantly after zeocin treatment (Fig. 4*A*), and consequently, the overall H3K27 methylation approached the WT level (Fig. 4*B* and Supplemental Table S4). In addition, zeocin caused a decrease in H3.3K36 methylation in *fas1* (Fig. 4*A*). Both H3.3K27 and K36 methylations were also affected by zeocin in the WT but to a lesser extent.

**Fig. 5:**
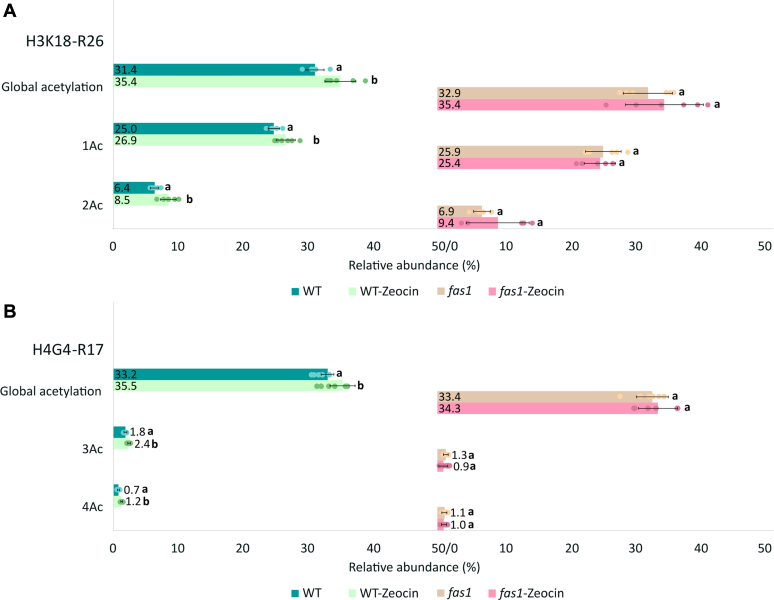
**The impact of zeocin treatment on H3 and H4 acetylation profiles.***A*, changes in global acetylation of H3K18–R26 peptide upon exposure to zeocin with a detailed view on identified forms showing that the levels of all H3K18–R26 forms (1ac–K18ac/K23ac; 2ac–K18acK23ac) were influenced by zeocin treatment in WT. *B*, changes in global acetylation of H4G4-R17 peptide upon exposure to zeocin with detailed view on increased levels of highly acetylated H4G4–R17 forms (3ac–K5acK8acK12ac/K5acK8acK16ac/K5acK12acK16ac/K8acK12acK16ac; 4ac–K5acK8acK12acK16ac) in WT. Numbers correspond to the mean values (n = 6) of precursor peak areas in percentages. For each histone form, letters a and b indicate significant differences between control (WT or *fas1*) and respective line treated with zeocin, according to the Student *t* test at *p* < 0.05. FAS, fasciata.

Conversely, we observed increased global acetylation of H3K18−R26 (**H3K18**QLATKAA**R26**) and H4G4−R17 (**H4G4**KGGKGLGKGGAK**R17**) in WT upon exposure to the genotoxic agent (Fig. 5 and Supplemental Table S4). Specifically, zeocin treatment increased the levels of monoacetylated (1ac; K18ac/K23ac) and diacetylated (2ac; K18acK23ac) forms, which was accompanied by a decrease in nonmodified forms of H3K18−R26. In case of histone H4, significantly increased levels of lower abundant triacetylated (3ac; K5acK8acK12ac/K5acK8acK16ac/K5acK12acK16ac/K8acK12acK16ac) and tetraacetylated (4ac; K5acK8acK12acK16ac) peptide forms were observed. Although the acetylation state of the *fas1* was not significantly affected by zeocin, a similar trend to WT was observed in terms of moderate increase in global acetylation of H3K18−R26. From the data obtained, we propose that H4 hyperacetylation in WT after zeocin treatment favors chromatin relaxation needed for DNA repair, while such an effect is weaker in *fas1* as chromatin structure is already opened due to a higher portion of H3.3 and H2A.W.7 variants. Relative abundance and *p* values of posttranslationally modified forms of histones H3.1, H3.3, and H4 are presented in Supplemental Table S4. We omitted the quantification of acetylated marks on H2A variants due to the low quality of the respective precursor peaks.

## Discussion

Genomic integrity is vital in regulating plant development under unfavorable environmental conditions. The regulation of key cellular events relies on chromatin remodeling, a dynamic process that is linked to epigenetic regulatory mechanisms such as DNA methylation, histone modifications, and histone variants. In this report, based on the proteomic study of plants with histone chaperone dysfunction, we suggest that maintaining genome stability *via* chromatin remodeling depends on a two-tiered process. The first level relies on a highly flexible mechanism that involves alterations in histone marks, while the second adaptive level requires the assistance of histone chaperones for the replacement of specific histone variants.

In-depth investigations of plant epigenetics in histone chaperone or chromatin remodeling mutants are rare (this work and (35)). Despite the substantial development of proteomic strategies, there is no universal approach for PTM characterization of all core histones and their variants, and the analytical setup has to be tailored to the main experimental objectives (45, 56). To investigate histone epigenetics during plant chromatin remodeling while reducing bias in the quantification of histone forms, we applied an MS-based approach, including chemical derivatization of amine groups with propionic anhydride (44). Although the differences in sample preparation, conditions of LC-MS/MS analysis, and data processing limit direct comparison of available datasets (this work; (35, 36)), we remark that the trends in the distribution of histone marks on the H3.1 and H3.3 are generally in agreement.

### Global Distribution of Histone Variants in WT and Histone Chaperone Mutants

In our previous study, we found distinct H3.3: H3.1 ratio in the *fas1 m123-2* and *fas1* mutants, accompanied by restored chromatin compaction and telomere and *rDNA* stability (20). Here, we extended this analysis to the level of noncanonical H2A variants. We found a significantly higher H2A.W.7: H2A.W.6 ratio in the *fas1* mutant line. H2A.W.6 represents the most abundant and highly expressed isoform in WT, as shown by us and others (10, 13). Observed changes in the H2A.W.7: H2A.W.6 ratio in *fas1* and *fas1 m123-2* plants seem to mimic the H3 variant profile. This indicates a compensatory mechanism for partial loss of H3.1, resulting in altered nucleosome composition. A similar model was suggested in *fas2*, where higher transcript levels of H2A.W variants and selected H3.3 like–genes were detected (10). Both H2A.W.6 and H2A.W.7 variants seem to participate in heterochromatin organization and be essential for heterochromatin accessibility (11). This is especially relevant for dynamic heterochromatin clusters, namely perinucleolar and intranucleolar clusters containing *rDNA*. Nucleolus contains highly transcribed *rDNA* organized into a complex structure, containing condensed sites enriched in H3.1 and H3.3 (or even repressive H3K9me2 mark) and less condensed H3.3 labeled foci (47, 57). We observed that the distribution of H2A.W.7 *in vivo* changes in the nuclei of *fas1* plants compared to the WT, with more frequent intranucleolar localization and a lower number of nuclei displaying pronounced chromocenters. Since the localization of H2A.W in *rDNA* has been established (14, 47), and this work), we suggest that the altered localization of H2A.W.7 in *fas1* protoplasts is likely associated with *rDNA* decondensation and its translocation from perinucleolar sites into the nucleolus, as described previously (58). This is consistent with the finding that the higher amount of H3.3 in the *fas1* line leads to a more open chromatin configuration, suggesting parallel functions of H2A.W and H3.3 in maintaining chromatin accessibility (11).

Another possible explanation for the altered ratio of H2A.W variants is related to the high instability of *rDNA* repeats in *fas1*, tied to a high frequency of DNA breaks. Recent studies have indicated a subfunctionalization of H2A.W.6 and H2A.W.7 variants, where the H2A.W.7 variant fulfills a role in DNA damage repair through the phosphorylation of the SQ motif (11). While H2A.W.7 acts during DNA repair in heterochromatin, similar role is assigned to H2A.X in euchromatin (14, 16). Nucleolar *rDNA* appears as a complex structure possibly involving both the heterochromatic islands intermingled by highly decondensed sites (47). As the accumulation of γH2A.X occurs in *fas1* and *fas2* nucleoli, it is possible that the enrichment of H2A.W.7 in the nucleolus relates to the increased occurrence of DNA breaks in *rDNA* in *fas1* (16, 21).

Phenotypic manifestations of *h2aZ* mutation in Arabidopsis increase with the number of knocked-out isoforms (7, 59). It has been documented that there is a dual functional role for the H2A.Z variant, one as a marker of the +1 nucleosome in expressed genes, the other as a repressive mark anticorrelated with H3.3 distribution in the gene bodies (4, 54, 60). Therefore, plants with a *H3.3* knockdown show increased H2A.Z occupancy on gene bodies (8). Our study reveals that the change in the H3.3: H3.1 ratio in *fas1* mutants does not result in alterations in the overall abundance of H2A.Z. This might relate to the fact that DNA methylation changes occurring in the H3.3 knockdown plants trigger changes in the H2A.Z occupancy (61).

It is currently unclear whether there is subfunctionalization of the H2A.Z isoforms, given their discrete roles at the transcription start site or the gene body. The only H2A.Z isoform that is expressed independently of the cell cycle is the dominant H2A.Z.9 isoform (62). NAP1 histone chaperone has been shown to have a minor effect on the H2A.Z deposition in promoter regions in yeast (52). We observed a decrease in the amount of H2A.Z.9 and increase in H2A.Z.11 levels in the *m123-2* plants when compared to WT, which indicates the involvement of the NAP1 chaperone complex in cell-cycle independent deposition of H2A.Z.9. Given that the incorporation of histone H2A variants might be directed by specific chromatin remodeling complexes (63, 64), it is not surprising that we did not see changes in the distribution of H2A.Z.8 or the interaction of H2A.Z.8 and H2A.Z.11 with the NAP1 chaperone in the *m123-2* and *fas1*, respectively. These changes might involve NRP1 and two that were shown to interact with H2A.Z and negatively affect their abundance in particular genomic sites and that act cooperatively with NAP1 in DNA repair (54, 65).

### Histone Modifications Distribution in WT and Chaperone Mutants

Histone H3 variants are an example of a functional specification. The canonical histone H3.1 is deposited to chromatin during the S-phase by CAF-1, while H3.3 histone shows replication-independent deposition, mediated by other histone chaperone complexes (66). The important difference in function between H3.1 and H3.3 histone variants is reflected in their PTM profiles. We observed a higher relative abundance of K27me1 in the H3.1 variant than H3.3. It has been previously reported that the enzyme responsible for H3K27me1, ATRX5/6, requires the H3.1A31 residue for its catalytic function (37), suggesting that epigenetic mitotically inherited memory mediated by H3K27 methylation occurs mainly through H3.1. On the other hand, activating methylation of lysine 36 (H3K36me2/H3K36me3) occurs predominantly on H3.3, as reported by (36). Furthermore, chromatin immunoprecipitation-seq studies of H3.1 and H3.3 deposition have shown that H3K9me2 is tightly associated with H3.1 enrichment (19), while H3.3 deposition correlates with active acetylation and methylation marks. This preferential modification of H3.3 with euchromatin marks seems to be a conserved feature, since it also occurs in insect or mammalian models (67, 68).

Our data indicate repurposing a subset of the H3.3 variant pool into a pseudo-H3.1 role in *fas1*. The significant relative reduction in H3.3K36 methylation status, together with the increase of H3.3K27me1 in the *fas1*, posits that H3.3 can partially substitute the role of H3.1 in the chromatin context, even though the kinetics of enzymes such as ATXR5/6 are suboptimal. The results from the study by Jamge *et al*. (35) are in agreement with the notion that both H3.1 and H3.3 are marked with both repressive and activating marks, albeit to a different extent, suggesting partial functional redundancy under stress conditions. Another important finding is that the proportion of unmodified H3.1K27-R40 peptides is lower, while unmodified H3.3K27-R40 is much higher in *fas1*. We hypothesize that this change might be caused by increased histone turnover, which could be the result of increased chromatin accessibility (20) or lower enzymatic activity of specific enzymes given by alanine to threonine replacement at the 31st position of H3.3 variant (37).

It has been previously suggested that the incorporation of H2A variants is crucial for discriminating the chromatin state of a particular genomic locus (35). The H3 methylation profile in *fas1 m123-2* mutants (lying between WT and *fas1*) may therefore indicate that the unbalanced H2A incorporation resulting from *Nap1* mutation affects the methylation on H3. These changes likely stem from alterations in chromatin states directed by the incorporation of the H2A variant.

### DNA Repair and Histone Modifications, a Complicated Relationship

Chromatin structure undergoes several changes during DNA repair–related remodeling since chromatin and bound proteins are thought to impede the efficient recognition and repair of DNA breaks. The current model for chromatin remodeling is known as the “access-repair-restore” model and involves transient periods of heterochromatinization, chromatin opening, repair, and restoration of the chromatin template (69). Chromatin at sites of DNA lesions is known to be transiently enriched in heterochromatin proteins (*e.g.*, like-heterochromatin protein 1) and marked by specific signaling marks (*e.g.*, H2A.X.K139ph and ubiquitination of H2A/H2A.X in the N terminus (70, 71). Subsequently, the chromatin template is made accessible through histone H4 hyperacetylation and incorporation of histone variants (*e.g.*, H2A.Z and H3.3; (72, 73)). Here, we observed significant changes at the level of histone PTMs but not in the ratios of histone H2A or H3 variants in WT plants exposed to zeocin. Our results show higher levels of acetylated H3K18−R26 and H4G4−R17 upon exposure to zeocin in WT, suggesting a transient opening of the chromatin template. Surprisingly, the absence of hyperacetylation on histone H4 and a further decrease in H3K36 methylation was observed in *fas1*. This pattern is consistent with a recent report showing the link between H3K36me3 and H4K5ac, which is mediated by NAP1 family proteins, involving both NAP1 and NRP1 (74, 75).

The impact of H3K36 monomethylation, dimethylation, and trimethylation is not fully understood in plants. In mammals, H3K36me3 relates to the ongoing transcription (3′terminus and gene body), while it associates with 5′ gene terminus in Arabidopsis, with the elongation process assigned to H3K36me2 (38, 76). H3K36 methylation is involved in various aspects of DNA repair; it triggers chromatin compaction in fission yeast (77), H3K36 methylation is upregulated during exposure to ionizing radiation in mammals (78), and recruits the DNA repair machinery. Decreased levels of H3K36 methylation are associated with lower DNA repair efficiency (79). Therefore, it remains an open question whether the reduced levels of H3K36 methylation in *fas1* relate to the DNA damage phenotype typical of *fas1* plants. The more open chromatin in the *fas1* determines the higher sensitivity of DNA to genotoxic stress and the acceleration of already ongoing compensatory and repair mechanisms due to easier accessibility to DNA lesions. It seems that the repair mechanisms in *fas1* are primarily directed to mimic the WT epigenetic state by balancing histone PTM status regardless of the presence of histone sequence variants. It is possible that chromatin carrying a higher level of the H3.3 variant is sufficiently accessible to allow DNA break repair and that further relaxation by hyperacetylation is not required. Since DNA breaks triggered by zeocin are stochastic by nature, likely occurring only in a subset of cells and affecting a minor portion of chromatin, it is also possible that global evaluation of histone variants and modifications by MS captures only changes that are highly abundant or spread around DNA lesions on chromatin (reported previously for H4 acetylation (80, 81)).

**Fig. 6:**
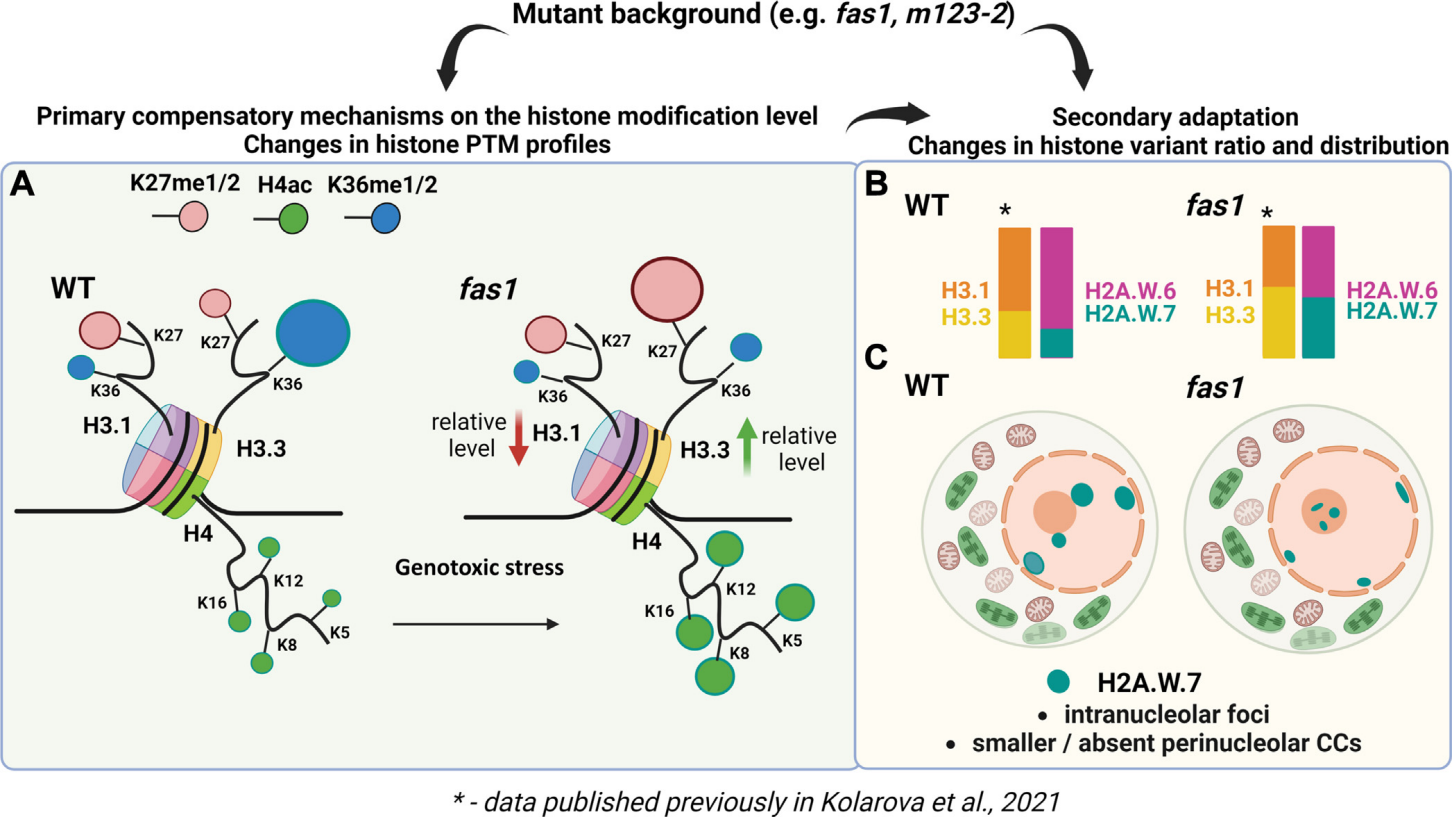
**Schematic representation of epigenetic changes in the *fas1* plants.***A*, changes in histone modification profiles, exemplified on H3.3 methylation on the K27–R40 peptide in WT and *fas1* (note: the homotypic/heterotypic nature of nucleosomes in terms of H3.1 and H3.3 is not reflected in the image). Changes in H4 acetylation status induced by the genotoxic agent are also depicted. *B*, histone variant changes including the upregulation of H3.3 (20) and H2A.W.7 in *fas1* compared to WT. *C*, relocalization of H2A.W.7 in the protoplasts of *fas1* plants, with higher frequency of H2A.W.7 localization in intranucleolar clusters and infrequent presence in nuclear chromocenters. Image created with Biorender.com. CC, chromocenter; FAS, fasciata.

Altogether, we show that MS is vital in studying the complex changes that are introduced in the histone chaperone mutant background, especially with other approaches that are complementary in their ability to map change in the distribution of histone variants or histone PTMs. We demonstrate that *fas1*, *m123-2*, and *fas1 m123-2* display numerous changes in the PTM profiles of histones, a possible primary mechanism for compensating the altered epigenetic landscape (Fig. 6*A*). Besides these rapid changes in the PTM profiles, the chaperone mutants show changes in the histone variant ratios and histone variant distribution (Fig. 6, *B* and *C*), which might be a slower adaptive process of mutant plants trying to cope with defective histone variant incorporation. This is also consistent with another observation (82), suggesting that plant responses to genotoxic agents primarily involve rapid repair mechanisms mediated by changes in PTM status rather than histone replacement. Future work will need to identify the downstream players of chromatin remodeling in the regulation of plant growth and development under genotoxic or environmental stress conditions to understand how the PTM status and composition of histone variants define the unique properties of nucleosomes.

## Data Availability

The MS proteomics data have been deposited to the ProteomeXchange Consortium *via* the PRIDE (83) partner repository with the dataset identifier PXD046034.

## Supplemental Data

This article contains Supplemental Data.

## Conflict of Interest

T. D. was employed by the company Labdeers. The remaining authors declare that the research was conducted in the absence of any commercial or financial relationships that could be construed as a potential conflict of interest.

## Supplementary Data

Supplemental Table S1 The list of the identified histone peptide forms by LC-MS/MS with the description of the associated proteins. Sheets 1 and 3 show Uniprot ID accession and protein description for each identified histone sequence variant with information about sequence coverage, number of identified peptides, PSMs and unique peptides, number of amino acids in the sequence, theoretical molecular weight and pI. Histone sequence variants quantified in the study are colored. Sheets 2 and 4 show modified peptides identified in each sample. The following parameters are present: Precursor Abundance, Ions Score, Expectation Value, Charge, Retention time [min], First Scan, Protein Accessions, m/z [Da], DeltaM [ppm],Deltam/z [Da].

Supplemental Table S2 The abundance of histone variants quantified in Skyline software. Sheet 1 shows absolute values of H2A.Z, H2A.X, and H2A.W variants in each replicate calculated from the abundance of unique peptides. Sheet 2 shows averaged relative abundance of histone variants in wt and mutant lines (data linked to Fig. 1) and results of statistical analysis (ANOVA followed by post hoc Tukey's HSD test). Sheet 3 shows averaged relative abundance of histone H2A and H3 variants in wt and mutant lines (data linked to Supplemental Fig. S3) and results of statistical analysis (ANOVA followed by post hoc Tukey's HSD test).

Supplemental Table S3 The abundance of histone H3.1, H3.3, and H4 post-translationally modified forms in wt, fas1, fas1 m123-2, and m123-2 plant lines as quantified in Skyline software. Sheet 1 shows absolute values of histone forms in each replicate. Sheet 2 shows percentage values and SD of histone forms and marks in each replicate (SDs represent error bars in Fig. 3, *A* and *B*). Sheet 3 shows averaged relative abundance of histone forms in wt and mutant lines (data linked to Fig. 3*A* are indicated in bold) and results of statistical analysis (ANOVA followed by post hoc Tukey's HSD test). Sheet 4 shows averaged relative abundance of K27 and K36 marks in H3K27–R40 sequences (histone marks of H3.1 and H3.3 were counted together; sum of histone marks equals 100%) in wt and mutant lines (data linked to Fig. 3*B*) and results of statistical analysis (ANOVA followed by post hoc Tukey's HSD test). Sheet 5 shows averaged relative abundance of global methylation in wt and mutant lines and results of statistical analysis (ANOVA followed by post hoc Tukey's HSD test).

Supplemental Table S4 The abundance of histone H3.1, H3.3, and H4 post-translationally modified forms in wt and fas1 plant lines after zeocin treatment as quantified in Skyline software. Sheet 1 shows absolute values of histone forms in each replicate. Sheet 2 shows shows percentage values and SD of histone forms and marks in each replicate (SDs represent error bars in Fig. 4, *A* and *B*). Sheet 3 shows averaged relative abundance of histone forms in wt and fas1 with and without zeocin treatment (data linked to Fig. 4*A* are indicated in bold) and results of statistical analysis (Student *t* test). Sheet 4 shows averaged relative abundance of K27 and K36 marks in H3K27–R40 sequences (histone marks of H3.1 and H3.3 were counted together; sum of histone marks equals 100%) in wt and fas1 with and without zeocin treatment (data linked to Fig. 4*B*) and results of statistical analysis (Student t-test). Sheet 5 shows averaged relative abundance of acetylation in wt and fas1 with and without zeocin treatment (data linked to Fig. 5) and results of statistical analysis (Student *t* test).
