# Supplementary material for: Histone Chaperone Deficiency in Arabidopsis Plants Triggers Adaptive Epigenetic Changes in Histone Variants and Modifications
Source: Mol Cell Proteomics. 2024 Jun 5;23(7):100795. doi: 10.1016/j.mcpro.2024.100795 (PMC11263794; doi:10.1016/j.mcpro.2024.100795)
Supplement: Supplemental data [file mmc5.pdf]

SUPPLEMENTAL DATA

Histone chaperone deficiency in Arabidopsis plants triggers adaptive epigenetic changes in histone variants and modifications

Michal Franek[1]†, Martina Nešpor Dadejová[1]†, Pavlína Pírek[1], Karolína Kryštofová[1,2], Tereza Dobisová[3], Zbyněk Zdráhal[1,2], Martina Dvořáčková[1,2]*, Gabriela Lochmanová[1,2]*

1. Mendel Center for Plant Genomics and Proteomics, Central European Institute of Technology, Brno, Czech Republic
2. National Centre for Biomolecular Research, Faculty of Science, Masaryk University, Brno, Czech Republic
3. Labdeers, Boskovice, Czech Republic

* Correspondence: gabriela.lochmanova@ceitec.muni.cz (G.L.),
orcid: 0000-0002-0490-9380
martina.dvorackova@ceitec.muni.cz (M.D.),
orcid: 0000-0001-5998-6159

† Equal contribution.

Running title: Epigenetic changes in chaperone-deficient Arabidopsis

SUPPLEMENTAL FIGURES

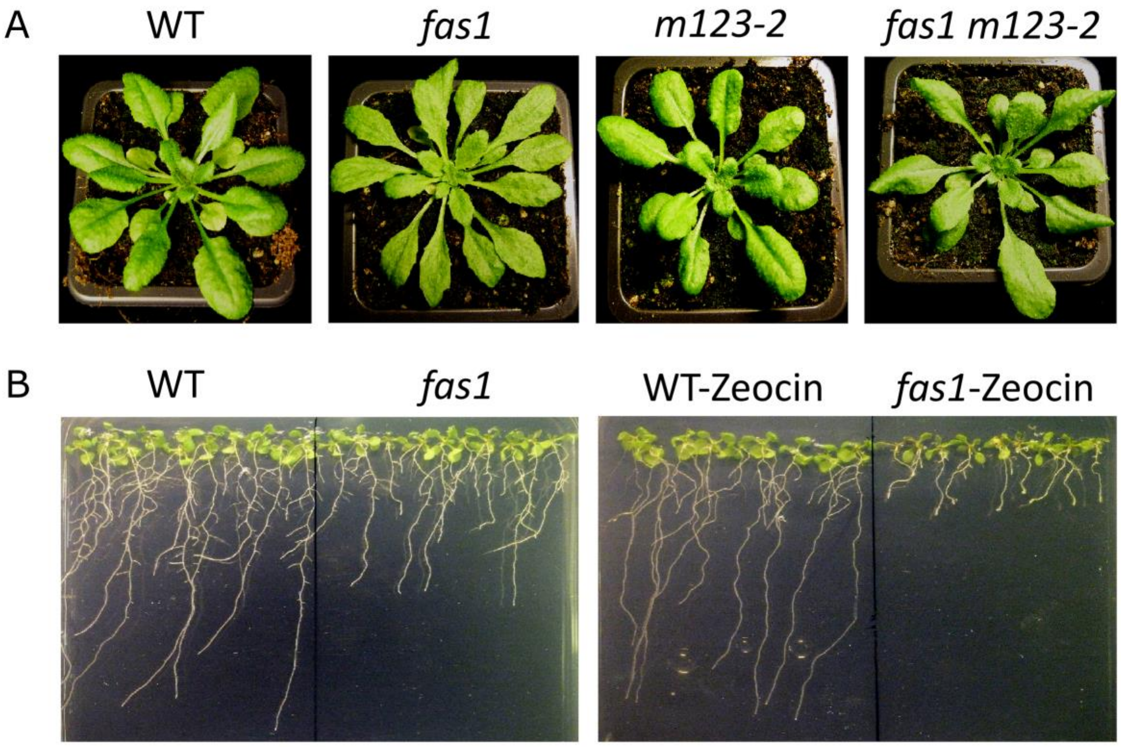

Fig. S1 Representative images of Arabidopsis wild-type and chaperone loss-of-function mutants. The typical *fas1* phenotype is pleiotropic and the most obvious defects include fasciated stems, dentate leaves, and short roots. *A,* Visible morphological changes are apparent in CAF-1-deficient mutant plants but not in *m123-2* plants. *B*, The impact of genotoxic agent zeocin on the phenotype of WT and *fas1* plants. After prolonged cultivation on zeocin (7 days), *fas1* plants exhibit significant growth delay compared to wild-type.

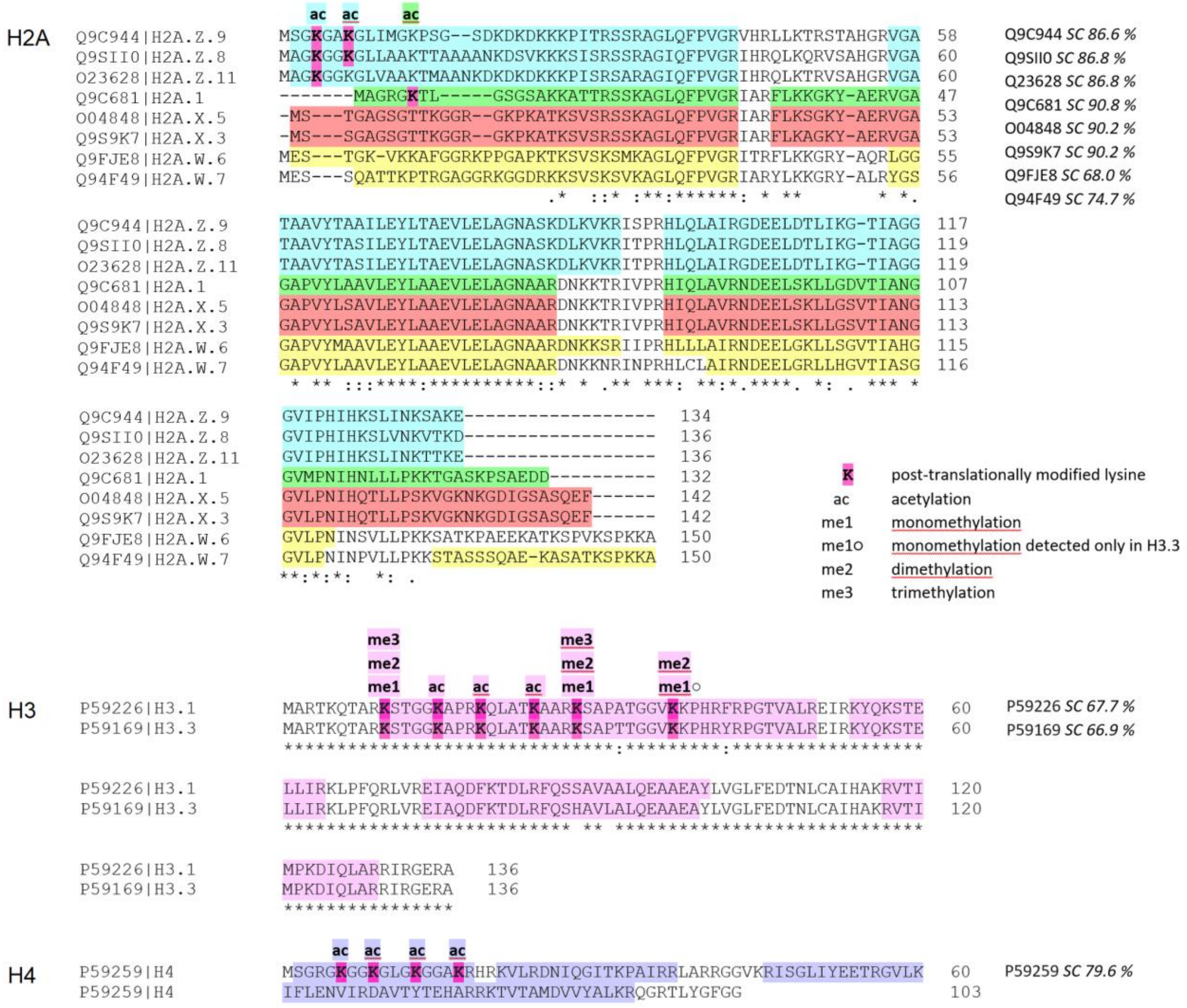

Fig. S2 The alignment of selected histone sequence variants identified in Arabidopsis wild-type and mutant lines using LC-MS/MS. Identified PTMs for each histone variant are indicated. Sequence coverage (SC) is shaded in colour and numerically indicated in the right.

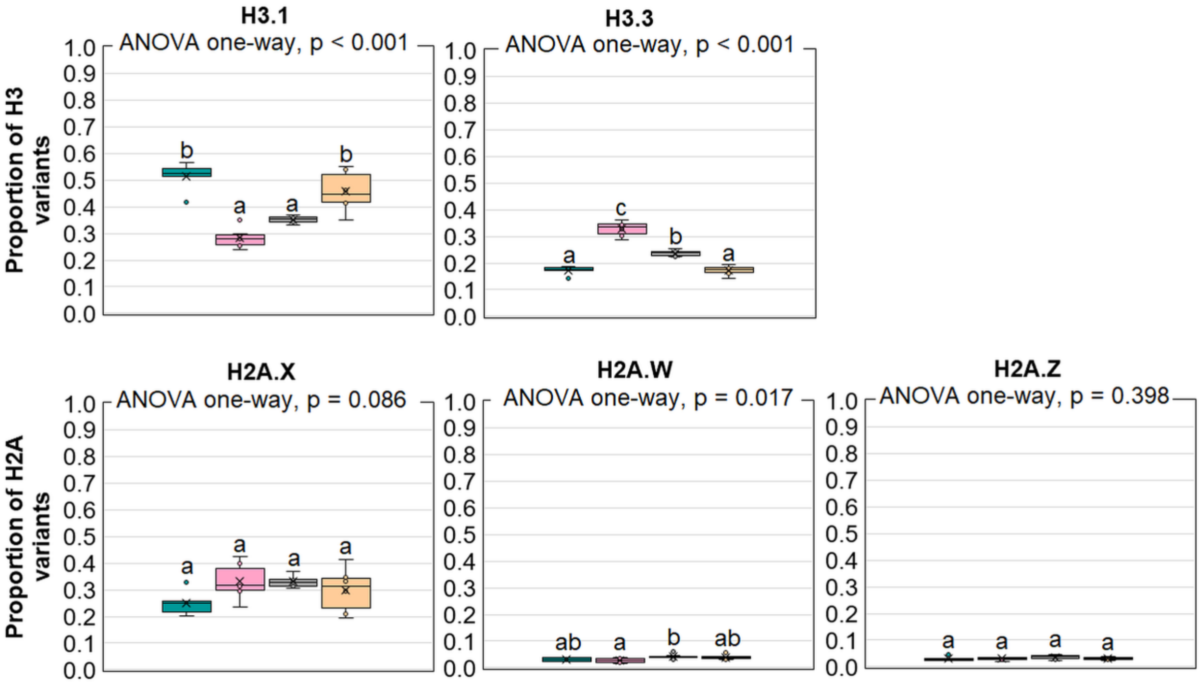

Fig. S3 The proportion of H2A and H3 variants in Arabidopsis mutant lines calculated from the intensities of variant-specific precursor peaks. For each histone variant, letters a - c indicate significant differences between WT, *fas1, fas1 m123-2,* and *m123-2* lines based on one-way analysis of variance (ANOVA) with post hoc Tukey's HSD test, $p < 0.05$.

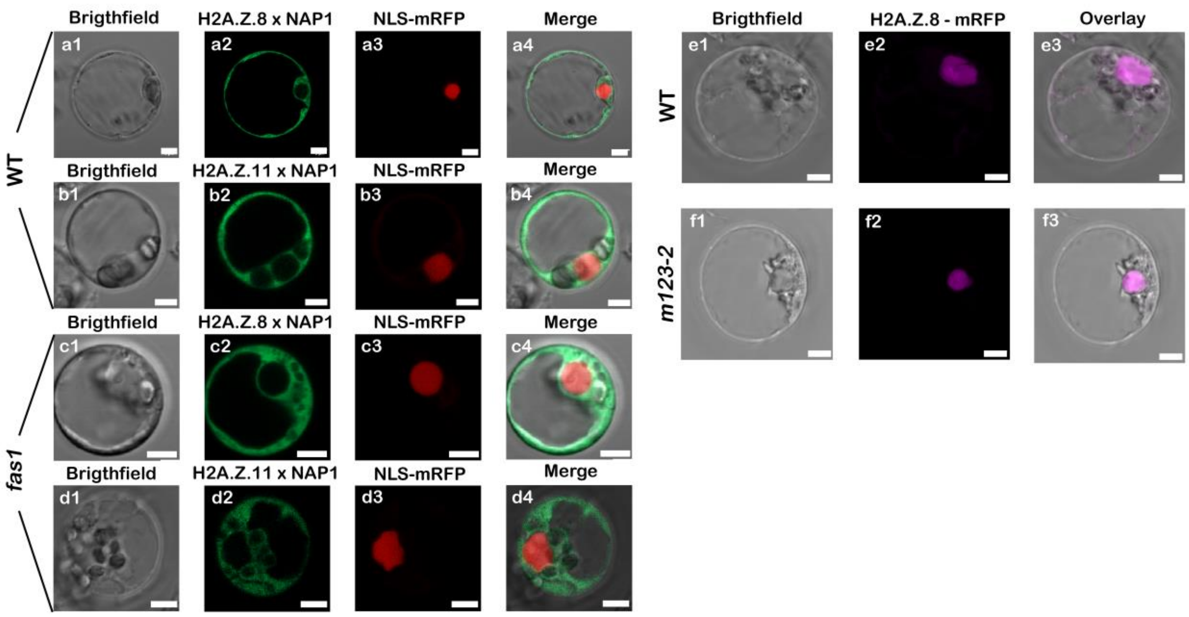

Fig. S4 Localization and interaction of H2A.Z with histone chaperone NAP1 in WT, *fas1* and *m123-2* lines. Cytoplasmic interaction between H2AZ.8 and the histone H2A/H2B chaperone NAP1 in wild-type (a, b) and *fas1* mutants (c, d). Brightfield images in a1 – d1, fluorescence complementation shown in (a2 – d2), with nucleus localization sequence in red (a3 – d3). Overlay images shown in (a4 – d4). The localization of H2A.Z8 is nuclear in wild-type (e1 – e3) and nap1 (f1 – f3) mutant plants. Scale bar – 5 µm.

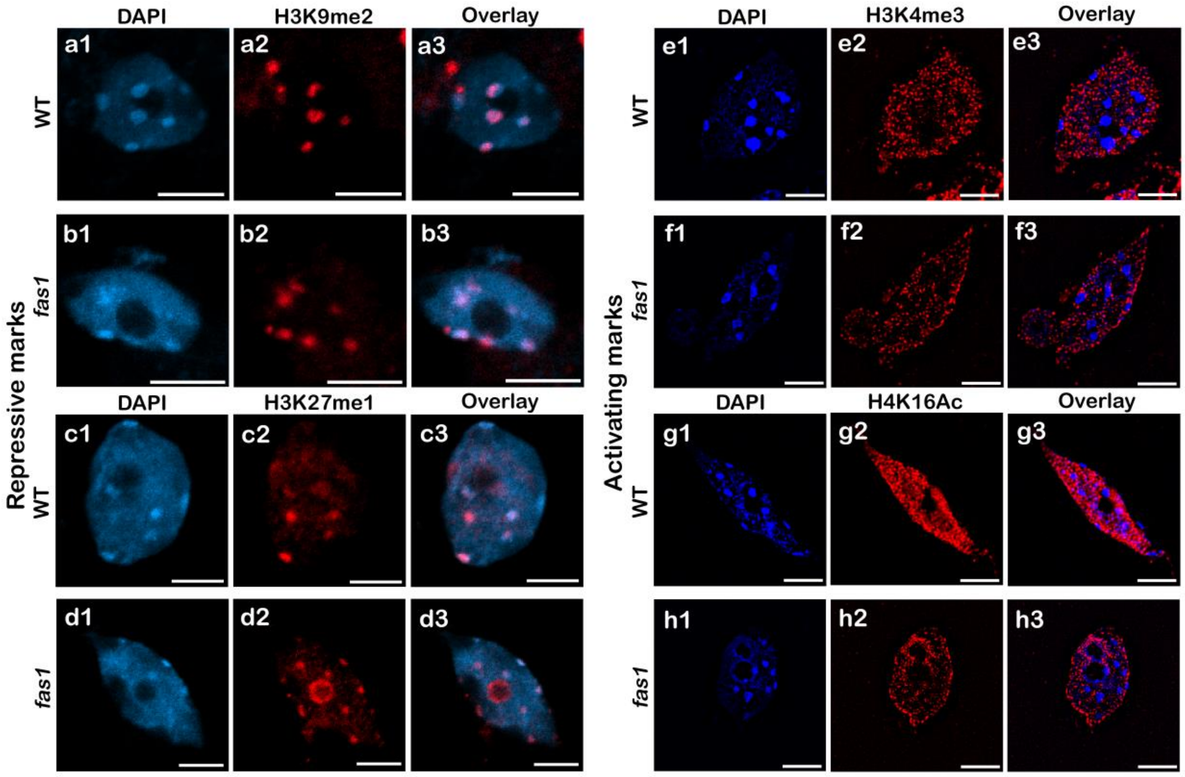

Fig. S5 Distribution of histone marks in the nuclei of wild-type and *fas1* plants. Distribution of repressive histone marks, such as H3K9me2 (a, b) and H3K27me1 (c, d) in the isolated nuclei of wild-type (a, c) and *fas1* mutants (b, d). Activating marks, such as H3K4me3 (e, f) and H4K16Ac (g, h) with a disperse nuclear pattern without clusters seen in both wild-type (e, g) and *fas1* plants (f, h). Scale bar – 5 µm.
